# Clinical and molecular characterization of isolated M1 disease in pediatric medulloblastoma: experience from the German HIT-MED studies

**DOI:** 10.1007/s11060-021-03913-5

**Published:** 2022-02-21

**Authors:** Denise Obrecht, Martin Mynarek, Christian Hagel, Robert Kwiecien, Michael Spohn, Michael Bockmayr, Brigitte Bison, Stefan M. Pfister, David T. W. Jones, Dominik Sturm, Andreas von Deimling, Felix Sahm, Katja von Hoff, B.-Ole Juhnke, Martin Benesch, Nicolas U. Gerber, Carsten Friedrich, André O. von Bueren, Rolf-Dieter Kortmann, Rudolf Schwarz, Torsten Pietsch, Gudrun Fleischhack, Ulrich Schüller, Stefan Rutkowski

**Affiliations:** 1 Pediatric Hematology and Oncology, University Medical Center Hamburg-Eppendorf, Martinistr. 52, 20246 Hamburg, Germany; 2 Institute of Neuropathology, University Medical Center Hamburg-Eppendorf, Hamburg, Germany; 3 Institute of Biostatistics and Clinical Research, University of Münster, Munster, Germany; 4 Research Institute Children’s Cancer Center Hamburg, Hamburg, Germany; 5 Department of Diagnostic and Interventional Neuroradiology, University Hospital Augsburg, Augsburg, Germany; 6 Hopp Children’s Cancer Center Heidelberg (KiTZ), Heidelberg, Germany; 7 Division of Pediatric Neurooncology, German Cancer Research Center (DKFZ), German Consortium for Translational Cancer Research (DKTK), Heidelberg, Germany; 8 KiTZ Clinical Trial Unit (ZIPO), Department of Pediatric Hematology and Oncology, Heidelberg University Hospital, Heidelberg, Germany; 9 Pediatric Glioma Research Group, German Cancer Research Center (DKFZ), Heidelberg, Germany; 10 Department of Neuropathology, University of Heidelberg, Heidelberg, Germany; 11 CCU Neuropathology, German Cancer Research Center (DKFZ), Heidelberg, Germany; 12 German Consortium for Translational Cancer Research (DKTK), Heidelberg, Germany; 13 Department of Neuropathology, University Hospital Heidelberg, Heidelberg, Germany; 14 Department of Pediatric Oncology and Hematology, Charité University Medicine, Berlin, Germany; 15 Division of Pediatric Hematology and Oncology, Department of Pediatrics and Adolescent Medicine, Medical University of Graz, Graz, Austria; 16 Department of Oncology, University Children’s Hospital, Zurich, Switzerland; 17 Department of Pediatric Oncology and Hematology, University Children’s Hospital Oldenburg, Oldenburg, Germany; 18 Division of Pediatric Hematology and Oncology, Department of Pediatrics, Obstetrics and Gynecology, University Hospital of Geneva, Geneva, Switzerland; 19 CANSEARCH Research Laboratory, Faculty of Medicine, University of Geneva, Geneva, Switzerland; 20 Department of Radiation Oncology, University of Leipzig, Leipzig, Germany; 21 Department for Radiotherapy, University Medical Center Hamburg-Eppendorf, Hamburg, Germany; 22 Institute of Neuropathology, Brain Tumor Reference Center of the German Society for Neuropathology and Neuroanatomy (DGNN), University of Bonn, DZNE German Center for Neurodegenerative Diseases, Bonn, Germany; 23 Pediatrics III, University Hospital of Essen, Essen, Germany; 24 Institute of Pathology, Charité University Medicine, Berlin, Germany

**Keywords:** Medulloblastoma, Children, Radiotherapy, Cerebrospinal fluid, Metastases

## Abstract

**Purpose:**

To evaluate the clinical impact of isolated spread of medulloblastoma cells into cerebrospinal fluid without additional macroscopic metastases (M1-only).

**Methods:**

The HIT-MED database was searched for pediatric patients with M1-only medulloblastoma diagnosed from 2000 to 2019. Corresponding clinical and molecular data was evaluated. Treatment was stratified by age and changed over time for older patients.

**Results:**

70 patients with centrally reviewed M1-only disease were identified. Clinical data was available for all and molecular data for 45/70 cases. 91% were non-WNT/non-SHH medulloblastoma (Grp3/4).

5-year PFS for 52 patients ≥ 4 years was 59.4 (± 7.1) %, receiving either upfront craniospinal irradiation (CSI) or SKK-sandwich chemotherapy (CT). Outcomes did not differ between these strategies (5-year PFS: CSI 61.7 ± 9.9%, SKK-CT 56.7 ± 6.1%). For patients < 4 years (n = 18), 5-year PFS was 50.0 (± 13.2) %. M1-persistence occurred exclusively using postoperative CT and was a strong negative predictive factor (p_PFS/OS_ < 0.01).

Patients with additional clinical or molecular high-risk (HR) characteristics had worse outcomes (5-year PFS 42.7 ± 10.6% vs. 64.0 ± 7.0%, p = 0.03). In n = 22 patients ≥ 4 years with full molecular information and without additional HR characteristics, risk classification by molecular subtyping had an effect on 5-year PFS (HR 16.7 ± 15.2%, SR 77.8 ± 13.9%; p = 0.01).

**Conclusions:**

Our results confirm that M1-only is a high-risk condition, and further underline the importance of CSF staging. Specific risk stratification of affected patients needs attention in future discussions for trials and treatment recommendations. Future patients without contraindications may benefit from upfront CSI by sparing risks related to higher cumulative CT applied in sandwich regimen.

**Supplementary Information:**

The online version contains supplementary material available at 10.1007/s11060-021-03913-5.

## Introduction

Medulloblastoma (MB) is one of the most common high-grade pediatric brain tumors [1]. Prognosis depends on clinical and biological risk factors [2–4]. Especially advances in the molecular characterization that led to the introduction of biologically defined MB subgroups and subtypes into the current WHO-classification are finding their ways into risk stratification for clinical trials [5–10].

Despite these advances, the extent of metastatic spread remains one of the key determinants for patients` outcomes [11]. Based on modifications of Chang`s staging criteria by identification of specific prognostic factors, metastases in MB are divided into four groups: microscopic metastases (M1) found only in cerebrospinal fluid (CSF), visible metastases in cerebral or spinal MRI (M2/3), or metastases outside the central nervous system (M4) [12–15]. Approximately one third of patients are expected to present with metastases at first diagnosis [16]. Optimal criteria for the definition of M1 are missing, therefore the actual proportion of M1-only cases is challenging to identify. Nevertheless, isolated microscopic CSF metastasis without neuroradiologically visible metastasis (M1-only) is a rare finding, that had been defined as a high-risk (HR) feature in the past by international consensus and is continously used by current trials and treatment recommendations [14, 17, 18].

To date, therapeutic recommendations are based on experiences from small patient numbers only [2, 18, 19]. In Europe, strategies using immediate postoperative radiotherapy (RT) and maintenance chemotherapy (MCT) as well as using postoperative chemotherapy (CT) followed by RT and MCT (“*sandwich strategies*”) are currently applied and have been changed during the last decades [11, 19, 20]. Although, in Germany, immediate postoperative RT using craniospinal irradiation (CSI) is the current standard of care based on the results from the HIT`91 and the HIT2000 trial, this will be changed by the beginning of the recruitment of the European-wide SIOP-E High-Risk Medulloblastoma (HR-MB) trial (EudraCT Number: 2018–004,250-17), where postoperative sandwich strategies will be further evaluated [11, 21]. In contrast, upfront RT is standard of care in North America and currently unquestioned [22].

Here, we aim to further characterize M1-only metastasis in pediatric MB by reporting our experience over the last two decades after the end of the HIT`91 trial.

## Patients and methods

### Patient selection

The HIT-MED database was searched for patients with MB and M1-only staging between August 1, 2000 and December 31, 2019, so patients from the HIT2000 trial (NCT00303810), the Interim-HIT2000 registry (NCT02238899) and the I-HIT-MED registry (NCT02417324) were considered for inclusion. All trials and registries were approved by the responsible ethics committees and informed consent was obtained in all cases. 1,547 patients were screened for extent of disease. Details of this study`s cohort are displayed in Table 1 and in the Supplement.

**Table 1:** Demographic details

	≥ 4 years (n = 52) [No. of patients (%)]		< 4 years (n = 18) [No. of patients]	
	Immediate postoperative RT (n = 26)	SKK-sandwich CT (n = 26)	SKK (n = 5)	Intensified Induction and CARBO/ETO-96 h (More intensive strategies) (n = 13)
*Sex* Male Female	15 (57.7%) 11 (42.3%)	21 (80.8%) 5 (19.2%)	4 1	9 4
*Trial/Registry* HIT2000 trial Interim-HIT2000 registry I-HIT-MED registry	10 (38.5%) 7 (26.9%) 9 (34.6%)	24 (92.3%) 0 2 (7.7%)	4 0 1	5 4 4
*Histologic subtype* CMB DMB LC/A-MB	23 (88.5%) 0 3 (11.5%)	23 (88.5%) 1 (3.8%) 2 (7.7%)	3 1 1	10 2 1
*Molecular subgroup* MB, Group 3 MB, Group 4 MB, SHH MB, WNT *not evaluated*	5 (25.0%) 14 (70.0%) 0 1 (5.0%) *6*	5 (35.7%) 7 (50.0%) 2 (14.3%) *(1* × *TP53 wildtype, 1* × *TP53 mutated)* 0 *12*	1 0 1 *(TP53-wildtype)* 0 *3*	7 2 0 0 *4*
*Molecular subtypes Gr.3/4* I II III IV V VI VII VIII low score *not evaluated*	0 0 4 1 4 0 3 4 0 *2*	0 3 2 0 0 1 2 4 0 *0*	0 0 0 0 0 0 0 0 1 *0*	0 0 1 3 1 1 0 0 2 *1*
*MYC-amplification* None *MYC* amplification *MYCN* amplification *not evaluated*	14 (82.3%) 1 (5.9%) 2 (11.8%) *9*	12 (80.0%) 1 (6.7%) 2 (13.3%) *11*	2 0 0 *3*	10 1 0 *2*
*Initial MRI-staging* M0/R0 (no residual tumor) M0/R < 1.5 cm^2^ M0/R ≥ 1.5 cm^2^	22 (84.6%) 4 (15.4%) 0	17 (65.4%) 8 (30.8%) 1 (3.8%)	4 0 1	7 4 2
*M1-persistence* Yes No *not evaluated*	0 16 (100%) *10*	5 (20.8%) 19 (79.2%) *2*	2 2 1	3 10 0

### Diagnostics

Patients were eligible if diagnosed with centrally reviewed neuropathologically confirmed MB, centrally reviewed CSF cytology confirming M1-disease, and centrally reviewed pre- and postoperative cranial MRI and spinal MRI confirming absence of M2/M3 metastases. CSF central review was performed according to Faltermeier [23]. M1 was defined as more than one tumor cell or any number of tumor cell clusters in a sufficient slide that was prepared from CSF obtained by lumbar puncture on postoperative day 14 or later, but prior to the start of the adjuvant therapy.

Response evaluation was performed via MRI and CSF and retrospectively assessed according to recommendations of RAPNO [24]. Central review has been recommended. After *complete remission* (CR) confirmed by central review, further central review was only performed at relapse suspicion.

M1-persistence was defined as persistent finding of tumor cells in lumbar or ventricular CSF after at least one therapy element (1 full CT cycle or full RT dose). M1-persistence without M2/3 was classified as *stable disease* (SD). M1-persistence in combination with any progressive MRI finding was classified as *progressive disease* (PD) and therefore included as an event into the survival analysis.

DNA methylation analysis was performed either retrospectively or as part of clinical staging routine on the Illumina 450 K or EPIC platforms using the Heidelberg Brain Tumor Classifier version 11b4 (www.molecularneuropathology.com). *MYC* and *MYCN* copy number was analyzed either by FISH, MLPA, or copy number profiles (CNV) derived from DNA methylation profiling. For this analysis, risk stratification according to MB subtype was performed using subtypes I-VIII as published by Sharma [25] and the Heidelberg Medulloblastoma Classifier version 1.0, retrospectively. Whole chromosomal aberrations (WCA) analogous to the definition of Goschzik [26] were retrospectively evaluated by analyzing CNV profiles.

### Treatment

**Fig. 1:**
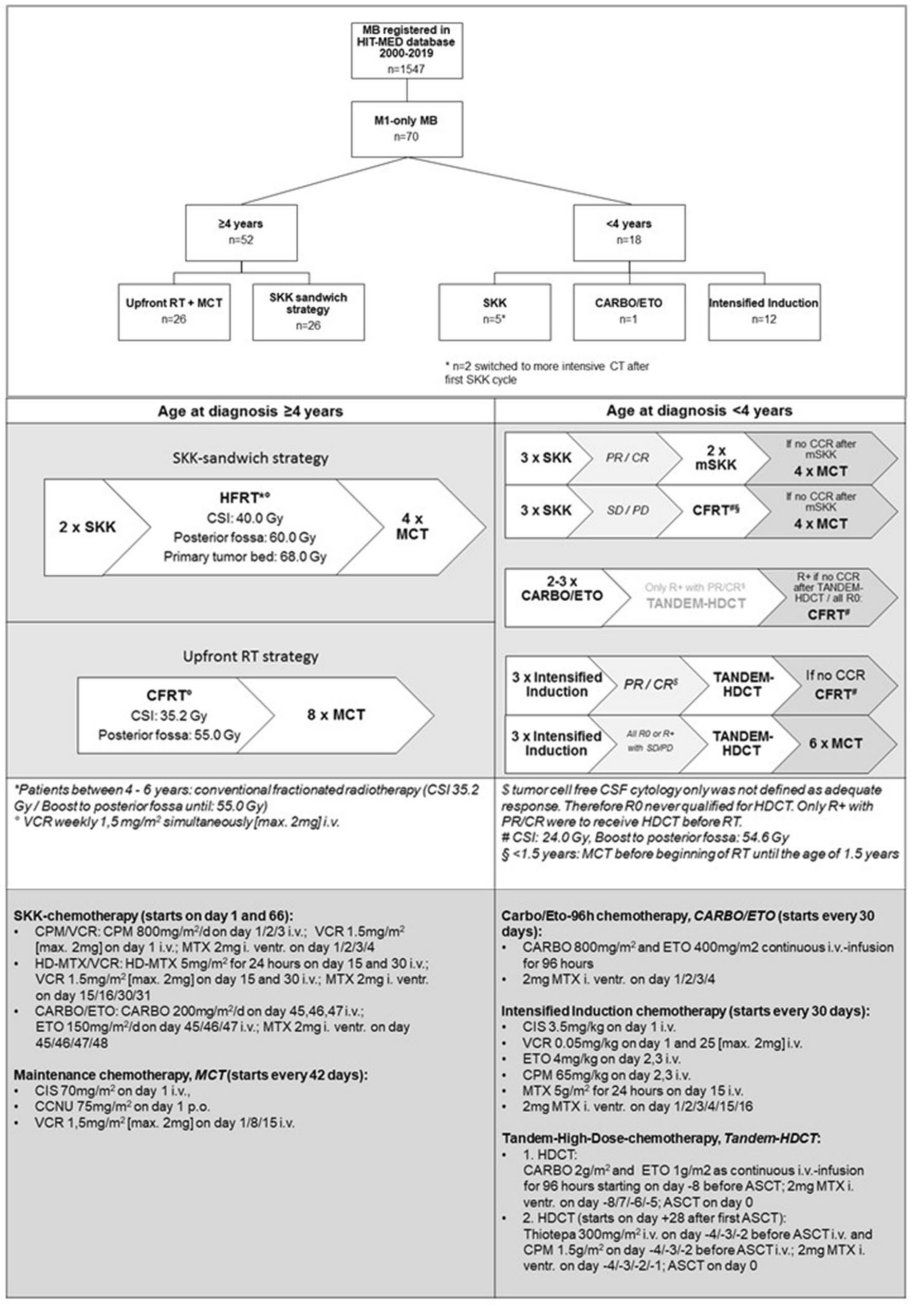
Consort diagram and treatment. Consort diagram showing the cohort identification process for n = 70 M1-only MB and their age-adapted therapy decision (stated treatment strategy according to first postoperative treatment element). The treatment overview below demonstrates the treatment strategies for patients ≥ 4 years at first surgery and for younger patients < 4 years during the observational time of the presented study. *Abbreviations:* MB: medulloblastoma, RT: radiotherapy, MCT: maintenance chemotherapy, SKK: chemotherapy for infant and young children (“*Säuglinge und Kleinkinder*”), mSKK: modified SKK, CSI: craniospinal irradiation, PR: partial response, (C)CR: (continuous) complete remission, SD: stable disease, PD: progressive disease, R + : residual tumor ≥ 1.5 cm^2^, CSF: cerebrospinal fluid, CPM: cyclophosphamide, VCR: vincristine, i.v.: intravenous, i. ventr.: intraventricular, (HD-)MTX: (high-dose) methothrexate, CARBO: carboplatin, ETO: etoposide, CIS: cisplatin, CCNU: lomustine, HDCT: high-dose chemotherapy, ASCT: autologous stem cell transplantation

Treatment stratification was based on central review staging results. Postoperative treatment is shown in Fig. 1. During the observational time of this study, for patients aged ≥ 4 years at first surgery, recommendation changed from SKK-sandwich strategy to upfront RT followed by MCT due to preliminary results on potential adverse outcomes of sandwich strategy in M1-only MB in the HIT2000 trial [19]. CSI dose was set to 40 Gy (hyperfractionated)/35.2 Gy (conventional fractionated) with boost to posterior fossa up to 60/55 Gy. In case of residual tumor, further boost to primary tumor bed up to 68 Gy was discussed. Patients aged < 4 years were treated with postoperative CT to avoid or postpone CSI. CSI dose was 24 Gy (conventional fractionated) with boost to the posterior fossa up to 54.6 Gy in young children.

### Statistics

Statistical analyses were performed using IBM^©^ SPSS® Version 27. Kaplan–Meier method was used for survival estimation. Log-rank testing was used to identify survival differences. Significance level was set to 0.05. All survival analyses regarding biological aspects including Cox regression were explorative. For allocation into sub-cohorts according to treatment, the first applied treatment element was used.

## Results

### Patients older than 4 years

52 of 1,183 (4.4%) registered patients fulfilled the inclusion criteria. Median age at diagnosis was 8.7 ± 3.6 (4.1–18.0) years. CSF was obtained 17.0 ± 6.2 (14–41) days after first surgery. 26 patients received upfront RT and SKK-sandwich CT, respectively. Median time to treatment was 38 ± 10 days for RT and 29 ± 10 days for SKK. Treatment delay more than 21, 40, and 49 days after tumor resection was not associated with inferior PFS. Five patients started treatment later than postoperative day 49 (3 × RT, 2 × SKK). Treatment modality was switched early to RT due to inadequate response to SKK in 4 cases. For SKK-treated patients, time to RT was 25 ± 6 (15–44) weeks.

Median follow-up for 34 surviving patients was 8.0 years (range: 1.2–15.9 years; RT: 5.8 ± 3.3 years; SKK: 11.9 ± 3.7 years; difference explained by the changed treatment recommendations during recruitment time). Regarding all patients, 5-year PFS and -OS were 59.4 ± 7.1% and 77.0 ± 6.1%, respectively. OS after 10 years was 55.4 ± 8.3%.

**Fig. 2:**
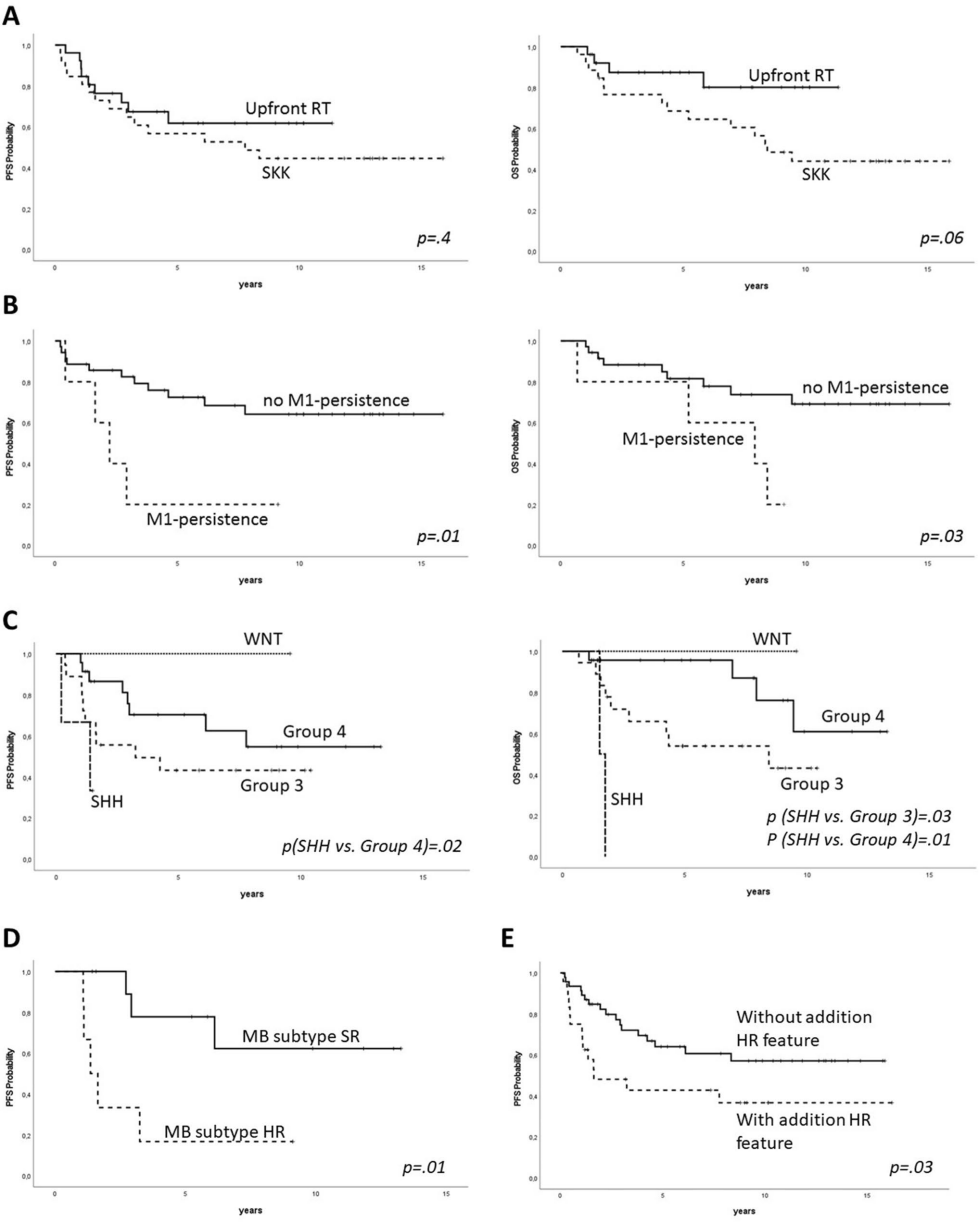
Kaplan Meier Plots. **A** PFS and OS according to treatment strategy in patients ≥ 4 years (n = 52). **B** PFS and OS according to presence of M1-persistence, ≥ 4 years (n = 40). **C**: PFS and OS according to MB subgroup, all ages (n = 45); p-value given for comparisons with p-value < .05. **D** PFS according to MB subtype, ≥ 4 years (n = 17). **E** PFS according to presence of additional HR-features (R > 1.5cm^2^/LCAMB/*MYC*-amplification/*MYCN* amplification in non-Group 4/HR according to MB subtype risk classification)

PFS and OS did not differ between the treatment groups (5-year PFS: RT 61.7 ± 9.9%, SKK 56.7 ± 6.1%, p_PFS_ = 0.4; 5-year OS: RT 87.4 ± 6.9%, SKK 68.5 ± 9.2%, p_OS_ = 0.06) [Fig. 2A]. The cumulative dose of intraventricular applied MTX during SKK (≥ 75% vs. < 75% of the scheduled dose [27]) was not associated with inferior outcomes (p_PFS_ = 0.9, p_OS_ = 1.0).

PD during primary therapy occurred in 11 cases (21.2%), and was equally distributed between the treatment groups (SKK: 6/26, RT: 5/26). During the observational time, 22 relapses occurred (local: 2, distant: 16, combined: 4).

### Patients younger than 4 years

4.9% of registered young patients presented with centrally reviewed M1-only MB at diagnosis. 5-year PFS and -OS for these n = 18 patients was 50.0(± 13.2) and 53.5(± 13.3) % with no significant difference compared to patients ≥ 4 years (p_PFS_ = 0.53; p_OS_ = 0.36). 5/18 patients received SKK-CT while 13/18 received more intensive CT as Intensified Induction or CARBO/ETO-96 h strategies (for details see Fig. 1). Within this small cohort, no statistically relevant difference between the treatment strategies was detected (5-year OS more intensive CT 61.5(± 15.3) %, SKK 30.0(± 23.9) %, p = 0.1; 5-year PFS more intensive CT 54.9(± 15.6) %, SKK 40.0(± 21.9) %, p = 0.3). Only 2 surviving young patients did not receive RT (1 × SHH-MB, 1 × CMB without molecular evaluation). One further patient (LCAMB) died without ever receiving RT due to fulminant relapse. RT-free survival in this cohort was 19.8(± 9.9) % after 10 years.

### CSF cytology during initial staging and treatment

Local interpretation of initial CSF cytology was reported for 46/70 cases. M1-positive CSF cytology was reported by local reviewer and confirmed by central review in 30 cases (65.2%). In addition, slides of 16 cases (34.8%) were falsely misinterpreted by local reviewers as M0 and rated as M1 by the central reviewer. In 24 (34.3%) cases, local CSF interpretation was not reported, but rated as M1 by central review.

During treatment, M1-persistence was a negative predictive factor (p < 0.01) [Fig. 2B] and exclusively occurred using postoperative CT [Table 1]. Tumor cells in the CSF were found in 5/15 evaluated relapsed cases ≥ 4 years. No isolated M1-relapse was detected.

### Biological characterization

Information on *MYC-* and *MYCN*-amplification status was available for 45/70 cases. *MYC* amplification was detected in 3 cases. *MYCN* amplification was present in 4 cases (all ≥ 4 years): 3 × MB Group 4 (1/3 died after relapse) and 1 × MB SHH (died after relapse).

Molecular MB subgroup was available for 45/70 patients [Table 1]. 91% (41 of 45 patients) were Group 3/4. 10-year PFS and OS for Group 3 was 43.2 ± 11.9 and 43.1 ± 13.6%, while 10-year PFS and OS for Group 4 was 54.4 ± 12.6 and 60.7 ± 17.1%, respectively, with two late relapses (laminar, located outside the posterior fossa) at 6 and 7 years after diagnosis in MB Group 4 [Fig. 2C]. Three cases of SHH-activated MB were detected. Two patients were ≥ 4 years of age at diagnosis. One was TP53-wildtype while the other showed TP53-mutation (somatic only). Both patients showed progression during primary treatment and died due to relapse. Another young patient with SHH-activated TP53-wildtype MB had no event reported, although follow-up was short (1.5 years). One patient with WNT-MB had no event.

Further analysis of patients with available molecular subgroup showed 33 (73.3%) males and 12 (26.7%) females, one patient with residual tumor ≥ 1.5cm^2^, 39 (86.7%) classic (CMB), 4 large-cell/anaplastic (LCAMB) and 2 desmoplastic (DMB) histology, 2 *MYC* and 4 *MYCN* amplified cases. 34 patients were ≥ 4 years at first surgery.

Non-WNT/non-SHH MB subtype (I – VIII) was available for 37 patients and showed low scores for 3 cases. In the remaining n = 34, all subtypes except for subtype I were represented in this cohort. High risk (HR: II, III and V) and standard risk (SR: IV, VI, VII, VIII) subtypes as defined by Sharma [25] were equally distributed among the treatment groups.

Copy number profiles from methylation arrays could be classified for WCA in 30/70 cases. For 7 patients with ≥ 2 WCAs, 5-year PFS and OS was 83.3 ± 15.2 and 100% while this was 56.5 ± 6.8 and 68.9 ± 6.3% for 0–1 WCAs (p = 0.11).

Including all these parameters into explorative Cox regression, *MYC* amplification was identified as only independent maker with impact on PFS (p < 0.01, hazard ratio 12.9). Risk classification by MB subtype did not have an impact on PFS regarding the whole cohort (p = 0.12).

### Separate analysis of cases with and without additional high-risk characteristics

**Fig. 3:**
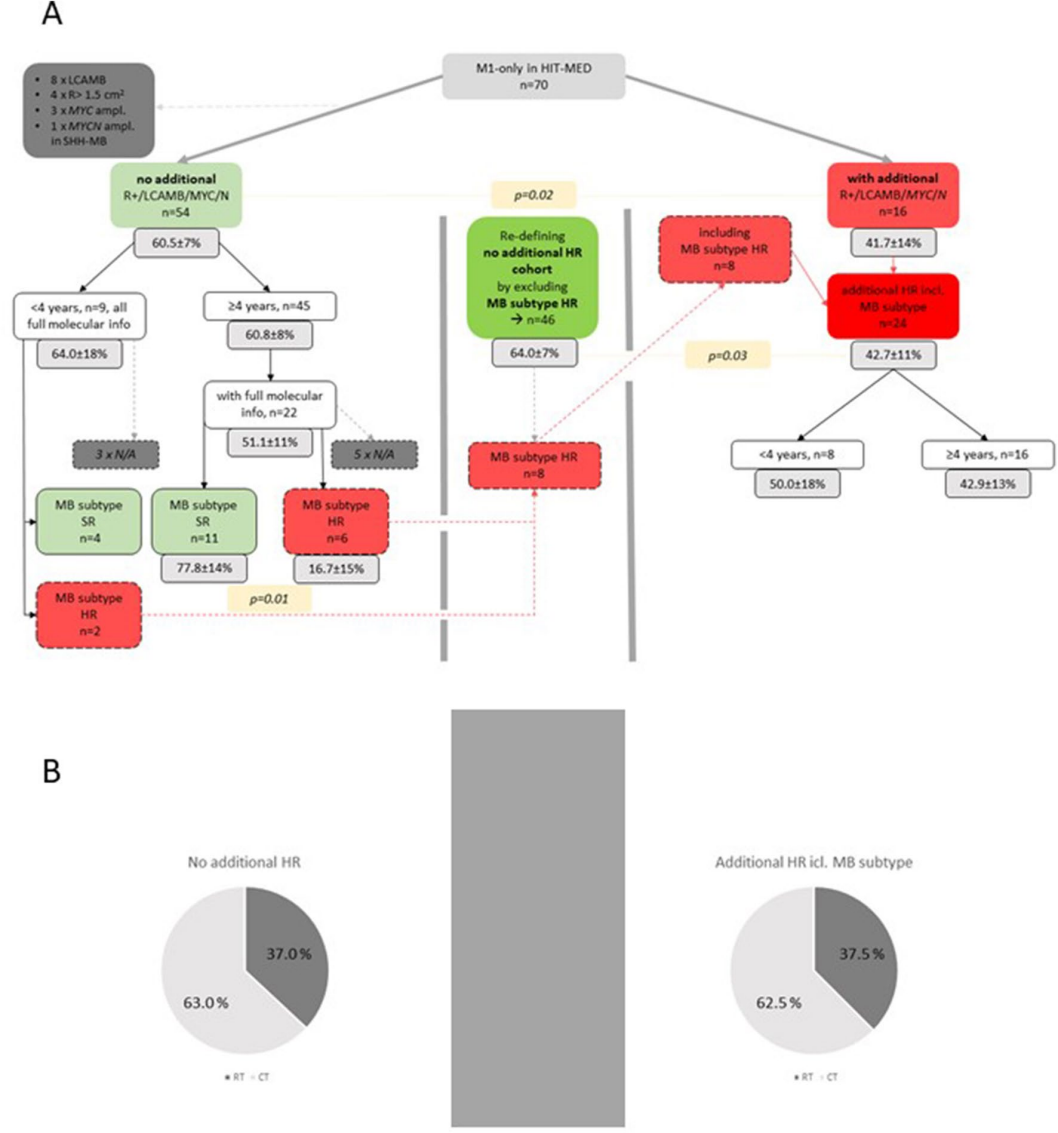
Overview of cohorts additional high-risk features and associated outcomes. **A** Columns beneath specific cohorts display 5-year PFS. P in columns is stated for the comparison of 5-year PFS of the linked cohorts. This figure gives an overview of patient`s additional risk factors and their impact on progression-free survival. First step was to divide the cohort by the presents of at least one of the following risk factors vs. no additional risk factors: LCAMB histology, presence of postoperative residual tumor ≥ 1.5cm2, presence of MYC/N amplification except MYCN in Group 4 MB. In the next step, patients without additional high-risk factors were subdivide by their age and molecular information was integrated to the model whenever available. Standard risk and high-risk allocation for molecular information was performed using MB subtypes as reported by Sharma et al. In case of HR-subtype, the case was re-allocated as case with additional high-risk feature (red dashed line), taking into account the significant inferior PFS of this sub-cohort, and cohorts “no additional high-risk feature” (green) vs. “additional high-risk feature” (red) were re-defined (middle column). **B** Diagram displays treatment strategy distribution among the identified cohorts: left “no additional high-risk features” based on n = 46 cases and right “additional high-risk features” based on n = 24 cases. For these diagrams all cases were included and MB subtype was respected whenever available

For 54 cases without additional HR characteristics (residual tumor > 1.5cm^2^, LCAMB, *MYC* amplification, *MYCN* amplification in other subgroups than Group 4), full molecular information was available for 22/45 patients ≥ 4 years and all nine patients < 4 years [Fig. 3]. In patients ≥ 4 years, molecular subgroup was Group 3 in 5, Group 4 in 15, SHH in 1 and WNT in 1 cases, respectively. MB subtype according to Sharma was HR in 6 and SR in 11 cases (II: 2, III: 2, IV: 1, V: 2, VI: 1, VII: 4, VIII: 5; not assessable: 5). *MYCN* amplification was detected in 3/22 MB Group 4. 5-year PFS for 22 cases ≥ 4 years was 51.5 ± 11.1% (5 events in RT cohort with n = 11, 6 events in CT cohort with n = 11). Risk classification by MB subtyping had an effect on outcomes in M1-only MB ≥ 4 years without additional risk factors in univariate setting (5-year PFS: HR 16.7 ± 15.2%, SR 77.8 ± 13.9%; p = 0.01) [Fig. 2D]. Multivariate Cox regression did not give valid results due to the limited number of cases. In patients < 4 years, molecular subgroup was Group 3 in 6, Group 4 in 2 and SHH in 1 cases. MB subtype was III in 1, IV in 3, V in 1, VI in 1 and not assessable for 3 cases. Out of these 9 cases 3 relapsed (5-year PFS: 64.0 ± 17.5%).

All patients with additional high-risk features other than M1-only (n = 24) were separately analyzed. 5-year PFS was 42.7 ± 10.6% and thus inferior to patients with M1 as only known high-risk characteristic as defined above (5-year PFS 64.0 ± 7.0%, p = 0.03) [Fig. 2E].

## Discussion

To date, M1-only disease in MB have mostly been reported in small numbers among larger MB series [14, 17, 18, 22]. Therefore, it`s frequency and clinical behavior are still not completely understood. The present study is based on a large multi-institutional cohort of pediatric patients with MB and M1-only, consistent central neuroradiological review, molecular information and standardized, but differing treatment strategies, and allows to further characterize the impact of M1-only. To our knowledge, this is the first MB series with prospective cytological CSF central review. The majority of cases reported here was part of the HIT2000 trial. Less patients from the registries qualified for inclusion into the present study due to the fact that the adherence to complete and sufficiently centrally reviewed staging was reduced in the registries. HIT`91 trial patients were not included into the present study, because central review processes and quality standards changed with introduction of the HIT2000 trial. Overall, the presented data decidedly extend the knowledge from earlier reports [11, 19].

Compared to general MB cohorts, CMB and LCAMB were more frequently found in this M1-only cohort, whereas DMB were less frequent [28]. Analyses for biological subgroups were explorative due to the limited number of cases. Most MB in this series belonged to Group 3 or Group 4. SHH- and WNT-activated MB were rare.

The presented data suggest that PFS and OS of patients with M1-only MB are inferior to non-metastatic MB (5-year OS: 80–91%) [11, 17, 29, 30], but might be superior to patients with M2/M3 at diagnosis (5-year OS: 35–59%) [11, 15, 17, 22]. This hypothesis is supported by the presented analysis for patients ≥ 4 years without additional high-risk characteristics showing a 5-year PFS of 64 ± 7%. These findings confirm that M1 itself is a high-risk feature and underlines the importance of CSF analyses during initial staging to enable reliable risk and treatment stratification. Further, they raise the discussion, if these patients need a distinct risk stratification from M0 and M2/3. Apart from this, we observed a relevant amount of event even after 5 years of surveillance leading to s relevant discrepancy between 5-years and 10-year OS and underlining the necessity of long-term follow-up. Furthermore, more than one third of local analyses of CSF in regard to the presence of tumor cells was falsely interpreted as negative but evaluated as positive by central review. This remarkable discrepancy between local and expert central review highlights the importance to implement central review processes keeping in mind that M1 is a high risk feature and influences treatment stratification.

Analyzing cases with and without additional established high-risk characteristics, we detected a clear difference in outcomes and affirm current consensus on established high-risk definitions. Notably, regarding pure M1-only MB without additional high-risk characteristics, risk stratification by MB subtypes as suggested by Sharma [25] led to identification of a high-risk cohort within patients without established high-risk characteristics other than M1. Therefore, we assume that MB subtyping may have an additional significant informative value also for high-risk medulloblastoma and needs to be evaluated in future trials.

Treatment strategies for the analyzed cohort included high-risk strategies with sandwich CT and postponed RT, which have also been used for children with macroscopic metastases (M2-M4) as well as immediate postoperative RT in patients ≥ 4 years. The longer interval until the radiotherapy start compared to chemotherapy in patients ≥ 4 years in the present cohort is explained by the more challenging and time consuming organization of RT. Nevertheless, the PNET4 trial reported a breaking point at postoperative day 49 for negative prognostic impact [30]. Here, we were not able to detect this effect probably due to the small number of patients. Furthermore, patients with delayed treatment start were equally distributed among the treatment groups.

The use of unchanged staging criteria enabled us to compare the effectiveness of upfront RT with SKK-sandwich CT in patients ≥ 4 years. In our series, the outcome of patients treated with upfront RT was comparable and not inferior to patients treated with sandwich CT. These results extend our previous observations where we reported even lower survival rates for M1-only MB after sandwich CT in the HIT2000 trial, compared to patients treated with upfront RT in HIT-91 (5-year OS for sandwich-CT vs RT: 31/81%) [11, 19]. Considering the main limitation of the presented study is the relatively small number of identified eligible patients, especially when analyzing sub-cohorts, statistical significance may not be detectable due to the sized of the cohort. For example, since survival curves display higher estimations for patients ≥ 4 years after immediate postoperative RT compared to SKK-sandwich CT and that this comparison almost reached statistical significance (p = 0.06), one can speculate that in a larger cohort immediate postoperative RT might have reached statistical significant superiority and is the overall superior treatment strategy. This speculation is supported by further arguments: Although all applied chemotherapy strategies contained intraventricular MTX as a direct treatment of the CSF in the whole neuroaxis, surprisingly, in this cohort, M1-persistence has been only observed using postoperative CT. Although speculative, this finding might be even more pronounced when CT regimens without intraventricular components are used. Considering M1-persistence was associated with poor survival, upfront RT may overall be superior to sandwich CT for RT-eligible M1-only MB. On the other hand, this effect appeared less relevant for patients without additional risk factors other than M1. Here, we observed almost equal outcomes for both treatment groups.

For patients not eligible for upfront RT, it remains to be clarified which CT strategy is the most favorable. Noteably, RT-free survival was alarmingly low in this cohort underlining the crucial role of RT in this context and the need for novel therapeutic strategies for this patient group.

Besides the limited patient number, several limitations apply for this study. First, CSF evaluation during treatment was recommended but not mandatory. Therefore, especially in the upfront RT cohort M1-persistence may have been missed.

Furthermore, the observational time for the upfront RT cohort was shorter than for the SKK cohort. Events may still occur in the future and data might be overinterpreted regarding the treatment strategy comparison.

Thus, performing postoperative CT is still reasonable to shorten the time to postoperative treatment in RT-eligible patients with iso-M1 MB, especially as bridging during staging processes with increasingly elaborate molecular tumor characterization as in the currently recruiting SIOPE HR-MB trial.

In conclusion, our results confirm that M1-only MB is a rare condition which needs further attention. These data suggest a distinct risk stratification for this cohort and underlines the importance of sufficient CSF staging and follow-up by expert central review. Our data suggests that for children eligible for CSI, immediate postoperative radiotherapy is probably at least as effective as sandwich CT strategies. Although this was not part of the investigations of this study, one can speculate that, with upfront RT, patients might benefit from less acute and long-term toxicities by sparing a relevant amount of cumulative systemic and even intraventricular chemotherapy depending on the national strategy. Here, further investigations are needed.

Especially during time consuming molecular tumor assessment, CT may function as a reasonable bridging method. MB subtyping has significant informative value in regard to risk stratification also in M1-only pediatric medulloblastoma.

## Supplementary Information

Below is the link to the electronic supplementary material.

Supplementary file1 (XLSX 14 KB)

## Data Availability

The study`s data can be found in the supplemental.xls-sheet.

## Declarations

### Research involving humans and/or animals

Consent of all participating individuals and/or their legal guardian was obtained.
